# VerSeDa: vertebrate secretome database

**DOI:** 10.1093/database/baw171

**Published:** 2017-02-24

**Authors:** Ana R. Cortazar, José A. Oguiza, Ana M. Aransay, José L. Lavín

**Affiliations:** 1 Genome Analysis Platform, CIC bioGUNE & CIBERehd, Bizkaia Technology Park, 48160 Derio, Spain; 2 Genetics and Microbiology Research Group, Department of Agrarian Production, Public University of Navarre, 31006 Pamplona, Spain

## Abstract

Based on the current tools, *de novo* secretome (full set of proteins secreted by an organism) prediction is a time consuming bioinformatic task that requires a multifactorial analysis in order to obtain reliable *in silico* predictions. Hence, to accelerate this process and offer researchers a reliable repository where secretome information can be obtained for vertebrates and model organisms, we have developed VerSeDa (Vertebrate Secretome Database). This freely available database stores information about proteins that are predicted to be secreted through the classical and non-classical mechanisms, for the wide range of vertebrate species deposited at the NCBI, UCSC and ENSEMBL sites. To our knowledge, VerSeDa is the only state-of-the-art database designed to store secretome data from multiple vertebrate genomes, thus, saving an important amount of time spent in the prediction of protein features that can be retrieved from this repository directly.

**Database URL:** VerSeDa is freely available at http://genomics.cicbiogune.es/VerSeDa/index.php

## Introduction

Secretome (full set of proteins secreted by an organism) analysis is currently a topic of interest in different fields such as cancer biomarkers identification (1), cell signaling in neurobiology, stem cells differentiation, thyroid hormone regulation (2), reverse vaccinology (3) or drug resistance (4). Accordingly, different studies have aimed to predict the secretome of a particular organism (5–7). Moreover, a detailed tissue-based map of the human proteome has been published recently where secreted proteins and membrane isoforms among the proteomes from 32 different tissues are catalogued. Data derived from this map are presented as an interactive resource by the Human Atlas Portal (8). At present, most of the existing tools for the study of secretomes are time-consuming and require knowledge of command line tools and programming skills. To overcome these possible limitations, we have recently developed different web-tools to enhance this kind of analyses through a user-friendly, one step analysis interface (9, 10).

At the moment, there is an earlier database on vertebrates secretomes called ‘SPD’ (11), which has not been updated since 2006 and includes just three species (human, rat and mouse). In addition, Meiken et al. (12) have recently published the database MetazSecKB that considers downloadable secretome predictions for multiple metazoan species, including a few vertebrates.

Herein, we present Vertebrate Secretome Database (VerSeDa) as the first database that stores information derived from the secretome analysis of all the predicted proteins from every vertebrate species available at the NCBI (http://www.ncbi.nlm.nih.gov/genome/browse/), University of California Santa Cruz (UCSC) (http://hgdownload.soe.ucsc.edu/downloads.html) and ENSEMBL (http://www.ensembl.org/index.html) databases, in addition to relevant model organisms’ proteomes. For VerSeDa, the prediction process was carried out through a well-established pipeline for classically secreted proteins prediction (13, 14). Moreover, this database is supported by predictions performed independently for each of the parameters, evaluated in the full analysis workflow. All those independent analyses were stored in this database and can be queried either to obtain information from each of the vertebrates and model organisms represented, or to test if a list of protein, gene or transcript IDs is predicted as secreted. Queries can be submitted in different formats and users can access the different parameters independently, in a batch, or following a workflow offered by the own database, making access to data intuitive through the web interface.

## Methods

**Figure 1.**
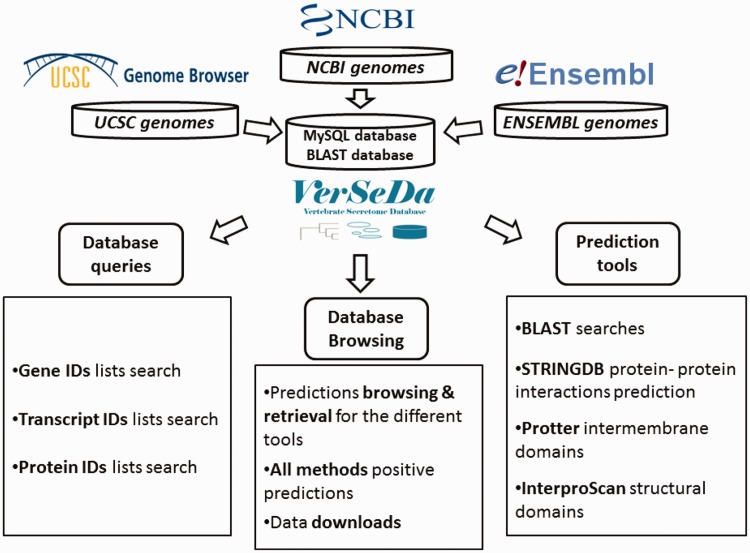
VerSeDa database structure.

To set up VerSeDa database’s structure (Figure 1), complete genome annotation sets from a total of 100 vertebrate and model organism species were downloaded from the links provided by the NCBI, UCSC and ENSEMBL repositories (see Supplementary Table S1 for the complete list of organisms and their genome version). Protein FASTA files were obtained for each of the genomes and, then, processed into our analysis pipeline as described previously in Cortazar et al. (10). VerSeDa is built on the predictions obtained from different publicly available software tools, namely, TargetP, SignalP (15, 16), PredGPI (17), TMHMM (18), WoLFPSORT (19), SecretomeP (20), arranged in a pipeline (10). The different tools are grouped in prediction steps and connected through the use of filtering scripts, so that predictions from one step are the input of the next. This technique allows discarding non-candidate sequences during the process, yielding a defined set of predictions at the end of the whole pipeline. Each tool was run with no cut-off limits on each organism full predicted proteome, to have the whole range of predictions for any protein annotated on those genomes, making those parameters available for consulting, simply interrogating the database. VerSeDa provides the user with additional features for each predicted protein of the retrieved list, explicitly, BLAST+ (21) searches against local database, InterproScan5 (22) for functional domains characterization, protein-protein interactions via STRINGDB API (23) and transmembrane graphical display using Protter (24) HTTP requests.

Predictions are indexed and queried via MySQL relational database with a MyISAM engine, improving results retrieval speed. VerSeDa is placed on a Linux/Apache server, for enhanced stability and security. Its architecture is built around a three-tier model: the presentation, the logic and the database tiers, providing this way scalable software, easy to maintain. The presentation tier handles user’s requests and displays data results and is implemented in PHP/CSS and JavaScript. The logic tier contains most of the functions to handle data transfer between the presentation and the database tier, and is implemented in Perl and R scripting languages.

Another important part of VerSeDa is the possibility of performing BLAST queries against its local BLASTdb of secreted proteins. To be considered by this tool, protein sequences must be first flagged as candidate for secretion. Therefore, we set the following cut-off scores for classical secretion predictions: SignalP and TargetP probability scores over 0.80, PredGPI *P*-value under 0.05, TMHMM detecting 1 or 0 transmembrane domains (TMD). The set of proteins yielded under those parameters is defined as ‘NonRefined Predictions Set’ (NRPS). To obtain a ‘Refined Predictions Set’ (RPS), we added WoLFPSORT to the pipeline keeping the ‘extracellular’ labeled predictions. Only proteins passing a cut-off score over 14 are retained. In both cases (NRPS and RPS), non-classical secretion predictions via SecretomeP with a cut-off over 0.9 are included accounting for a set of ‘Non-Classically’ (not signal peptide triggered) secreted proteins pool. In order to curate the protein records, we integrate UniProt (25) curated protein databases’ results in VerSeda, to select only those that are labeled as secreted, thus, obtaining two new categories ‘NonRefined Predictions Curated Set’ (NRPCS) and ‘Refined Predictions Curated Set’ (RPCS). The four sets are available as BLASTable databases.

## Results

This database’s workflow intends to simplify access to secretion related features from proteins, allowing browsing by customizable query parameters or the direct submission of protein ID lists to question the database directly, instead of submitting full sequences to prediction tools and having to wait for individual results.

VerSeDa website is divided into six individual pages: Homepage, Database browser (Organism), Database search (User query), BLAST search, Downloads and ‘Help & FAQs’. All those HTML documents are accessible from the interface’s main menu at the top-left corner. The Homepage offers an introduction to the database’s foundations and the kind of data stored. ‘Help & FAQs’ is a detailed user’s guide that explains how to browse and make use of the different VerSeDa’s data retrieval options as well as the Download and BLAST tool functionalities. Further details for each of the query options available and the local database BLAST tool are explained in their particular sections later in this manuscript.

## Investigating full genomes: database browse (organism)

As introduction to this interface (Supplementary Figure S1), there is a brief description of the different suitable queries. In this particular case, different filtering parameters are available. Eligible organism and cut-off values for each prediction tool (SignalP, TargetP, PredGPI, TMHMM, WoLF PSORT and SecretomeP) can be customized by the user or left on default values. Also, instead of selecting individual filters and their corresponding thresholds, a query that considers all the established parameters and default values is available as ‘Full search’ option, allowing to predict all the proteins that are considered as extracellular, under that cut-off scores, in a single step with or without curation according to UniProt database. Besides, structural domain screening is also available as optional analysis. Therefore, database searches facilitate the retrieval of the full set of proteins predicted as secreted from each included organism.

VerSeDa browser’s results appear as a table (Supplementary Figure S2) where Protein, Gene, and Transcript IDs are depicted in the first three columns and, in case that there is a UniProt identifier available, it is shown in the last column of the table. Other columns of the table correspond to each of the prediction tools’ values mentioned before. Clicking the ENSEMBL or Refseq protein ID (depending on each organism available IDs) redirects to an individual page (Supplementary Figure S3) for the selected protein, where different pieces of data related to it are displayed: gene and transcript sequence IDs in other databases, parameters obtained for each prediction tool, UniProt data curation, InterPro structural domain predictions with GO IDs, graphical representation of the structural domains, its FASTA sequence, a link to Protter intra-membrane domain structure and STRINGDB protein–protein interaction predictions (if available).

Downloadable results can be retrieved in different formats. Options are: ‘ID List’, which yields a simple list of the proteins predicted as secreted for the chosen organism with selected cut-off values, ‘FASTA file’ that returns the predicted proteins in FASTA format, and ‘Both’, the combination of previous options. Apart from the ID list and/or the FASTA file output selected, a results table equivalent to the one displayed online is added to the downloadable results folder along with a table containing the structural domains information, if that option was chosen by the user. In addition, a text file indicating the cut-off values of the query is incorporated. Moreover, individual predicted proteins information (in a zip file) can also be downloaded through the corresponding link on its respective pages. Finally, the ‘Submission’ option enables the input of an e-mail address to receive a link to the results after completion.

## Customizing research modes: database search (custom query)

As in the previous form, a paragraph introduces the interface (Supplementary Figure S4) and provides a brief description of available search options. In this section, the selection of the organism and the IDs type used for to query the database (gene, protein or transcript IDs) are mandatory. After that, the list of protein identifiers subject of analysis can be pasted into the text box and submitted to elucidate if they are characterized or not as secreted proteins. Another option is to directly upload a file with a list of identifiers, one per line. These identifiers admit different repositories ID types, depending on the selected organism. Analogous to Database browse functionality, cut-off values for the prediction tools can be customized by the user or left on default values. The ‘Full search’ option, previously described to predict all extracellular proteins, is also accessible in this form. Consulting the database by this method allows making predictions on lists of IDs (including just the sequences of interest), avoiding the retrieval of the protein FASTA sequence of the full predicted secretome. Extracting defined sets of candidates enables the direct use of these sequences for further processing (e.g. submission to prediction tools, results filtering and other characterizations).

The result yielded by this option is a table analogous to the table presented on the results’ page with the data of all the proteins queried by the user, a list of the predicted IDs, the user’s input list (for verification), a file summarizing the selected cut-off values and a FASTA file with the sequences of the interrogated proteins. If the structural domain screening is performed, a file including that information is appended to the output. Each entry of this table is also connected to the individual protein result page (Supplementary Figure S3). In this case, users can also provide an e-mail address to receive a URL for retrieving their results after completion.

## Looking for similarities: BLAST search

BLASTp, for seeking out protein databases using a protein query, and BLASTx, which searches protein databases using a translated nucleotide query, are available as complementary tools in this database (Supplementary Figure S5), enabling to scan amino acid or nucleotide sequences against a local BLAST database (BLASTdb), which is built upon protein sequences predicted as secreted among the organisms surveyed to develop VerSeDa. This way, users can compare their own query sequences against four databases (NRPS, RPS, NRPCS and RPCS) to find orthologs or simply retrieve reliable information from pairwise comparisons between their sequences and VerSeDa’s.

Local BLAST searches yield a regular report in blast output format 7 (plain text, tab separated format, representing the hits detected for each sequence submitted one per line) as described in the manual (http://www.ncbi.nlm.nih.gov/books/NBK279675/). Results are displayed in the webpage and are also available for downloading. The e-mail address option to receive a link to the results is also enabled for this tool.

## Retrieving bulk datasets: downloads

A downloadable compressed file, including sequence files from the different pools of proteins predicted as secreted (NRPS, RPS, NRPCS and RPCS) for any of the organisms in the database, is implemented here (Supplementary Figure S6), allowing users to download individual genome sets or bulk database predictions to enhance data retrieval.

## Discussion and conclusions

This database is meant to be a repository for secreted proteins obtained through the *insilico* screening of the predicted proteomes from vertebrates and other model organisms available at the NCBI, UCSC and ENSEMBL web-servers. VerSeDa comprehends predictions made by different bioinformatic tools arranged in a well-established pipeline (9, 10) to enhance the quality of the sequences incorporated to the database’s repositories, in addition to the integration of data retrieved from UniProt database to indicate which of the predictions can be determined as curated. This particular design improves the whole analysis speed and reliability. SQL indexing grants user the access to either a full organism proteome (searching for proteins passing the cut-off parameters defined by the user) or submit a list of protein IDs from an organism to revise which of them can be listed as secreted with a high improvement in the speed of results retrieval when compared to a complete secretome prediction analysis. Other analyses like BLAST alignments against the different database repositories (NRPS, RPS, NRPCS and RPCS) are also available. In addition, results yielded by this database are structured in multiple layers, so that, different information levels about selected proteins (from structural domains to protein-protein interactions) are accessible online and also in plain text format tables (available for downloading), thus, enabling users to retrieve results for post-processing at their local machines.

There are other databases built upon secretome predictions (26) that grant access to annotated lists of proteins, for instance, the repository of fungal predicted secretomes FunSecKB2 (27), their plant counterpart PlantSecKB (28), the comparative genomics oriented LAB-Secretome (29), which is aimed to lactic acid bacteria secreted proteins or MetazSecKB (12) that includes multiple metazoan genomes and stores lists of proteins’ predictions related to their subcellular location (secretion included). VerSeDa, on the other hand, is targeted to vertebrates (as core of the database), allowing queries on metazoans (e.g. human, mouse, frog or zebra fish) and a selection of relevant model organisms (e.g. fly, frog or worm). The main advantage of VerSeDa is that in addition to allowing users to download pre-computed predictions from the database, as in the case of the previously mentioned repositories, we have gone one step further taking advantage of the broad range of query parameters supported, enabling their customization and, therefore, the retrieval of diverse sets of proteins depending on the parameters chosen. Furthermore, classical and non-classical (leaderless secreted proteins) secretome predictions are archived in our collections differing from MetazSecKB, which relies exclusively on proteins from the classical pathway.

Access to descriptive reports for each of the putatively secreted proteins is supported as well, aiming to offer further details on protein features preventing multiple searches at particular databases. The purpose of this database is to keep up-to-date with new vertebrate genome releases and updated versions of the genomes already stored, being a reliable website to find information on the current state of secretome predictions in an increasing number of genome sequences/projects. Updated genomes will be managed via programmatic access through API connections to NCBI, UCSC and ENSEMBL. Subsequently, new organism sequences will be downloaded, processed by VerSeDa’s prediction tools, compared with UNIPROT curations if available, and results will be included in the database. Furthermore, we aim to include users’ favorite organism proteome to be analyzed and update that info. As well as manually curated sets of secreted proteins, in favor of improving our database’s contents. Those contributions can be submitted through the webpage’s Contributions form. Once reviewed by our team, curated tables will be added to VerSeDa. Every submitter will be acknowledged for contributing to this repository.

## Supplementary Material

Supplementary Data
