# Supplementary material for: VerSeDa: vertebrate secretome database
Source: Database (Oxford). 2017 Feb 24;2017:baw171. doi: 10.1093/database/baw171 (PMC5467544; doi:10.1093/database/baw171)
Supplement: Supplementary Data [file baw171_Supp.docx]

**Figure S1.** Full Organism data browse page


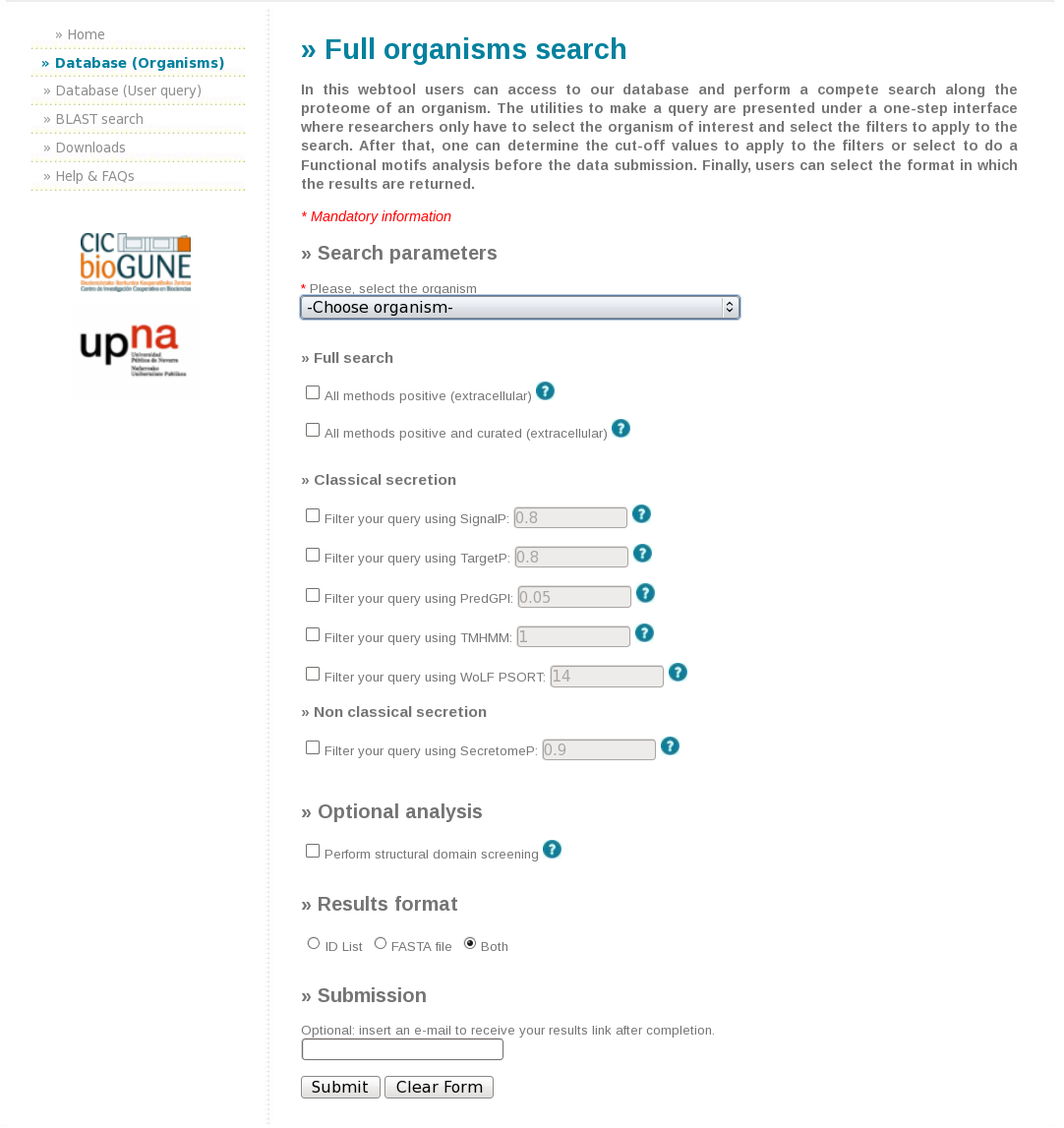


**Figure S2.** Results table example


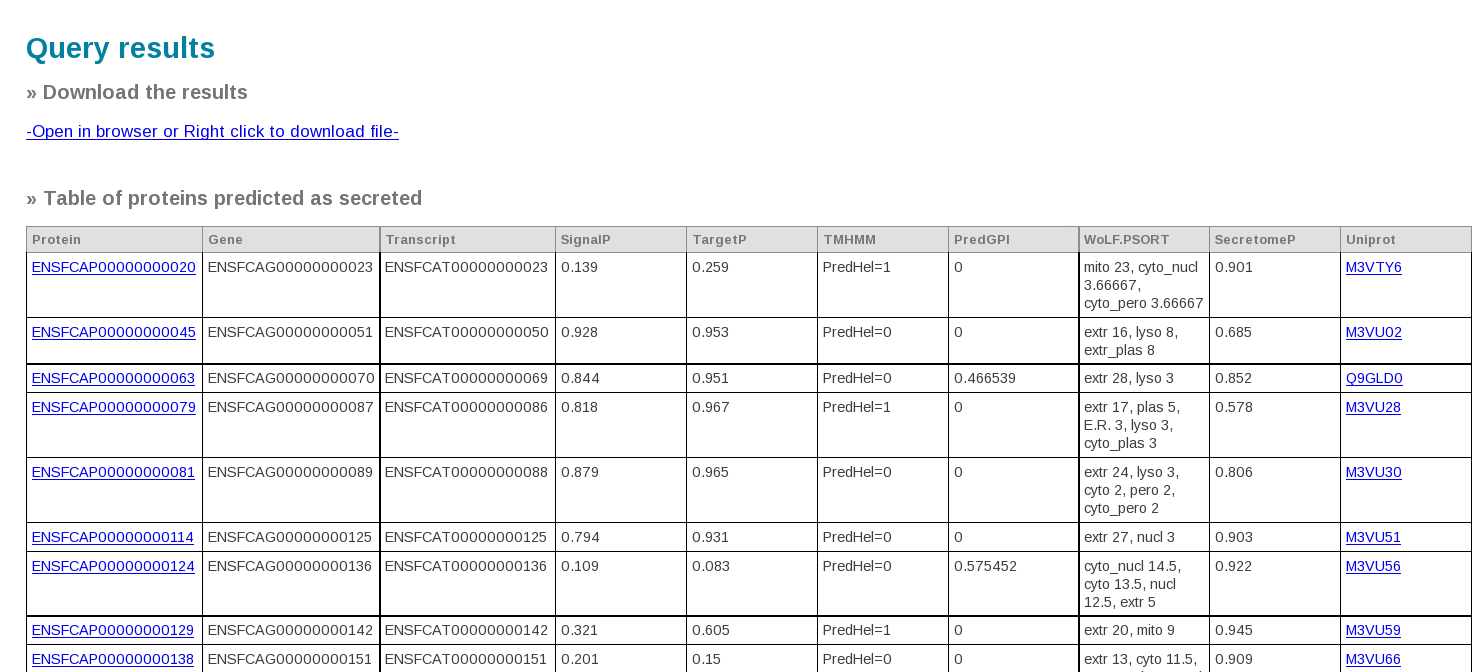


**Figure S3.** Individual protein results page

**
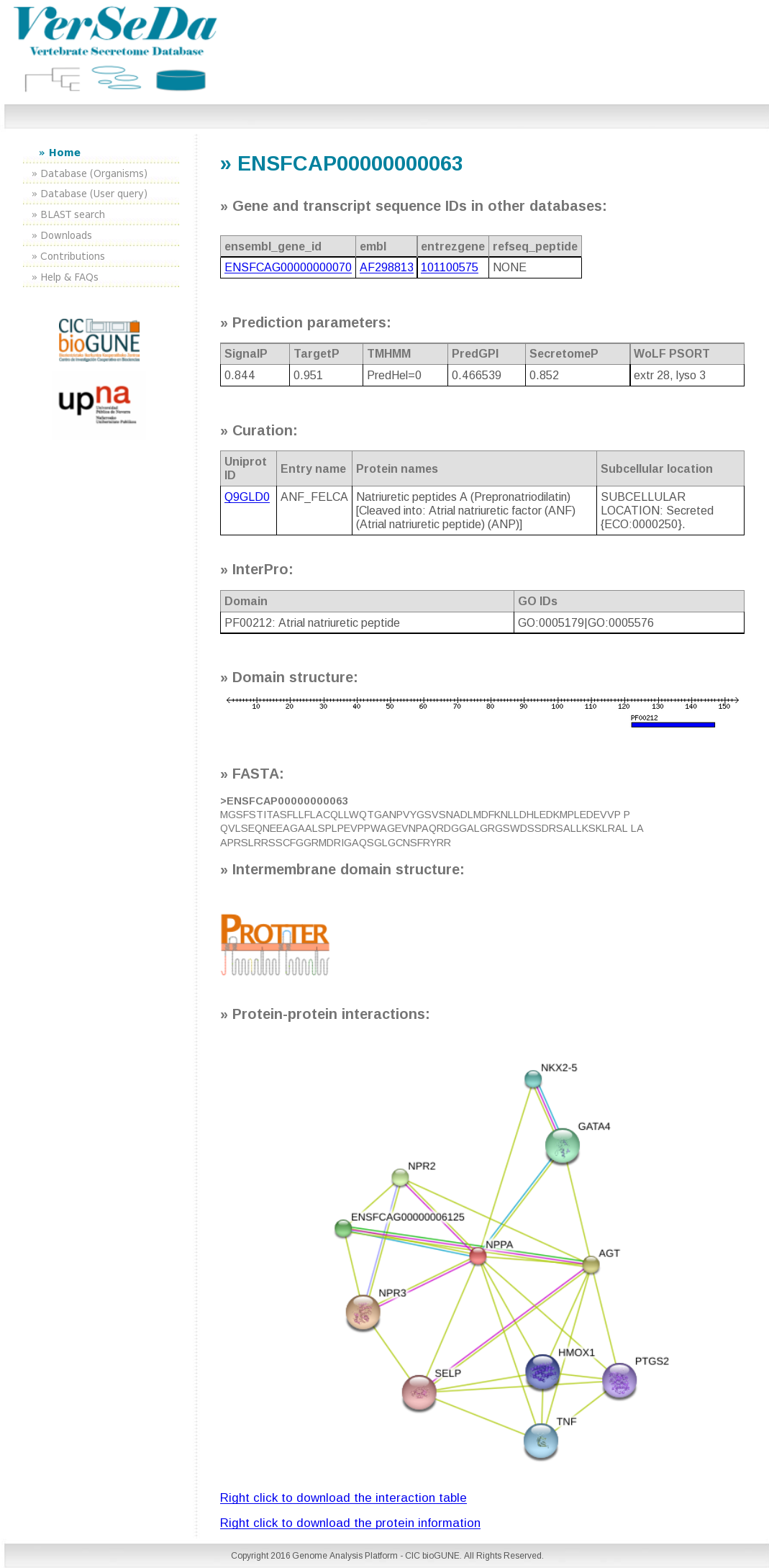
**

**Figure S4.** User Query interface

**
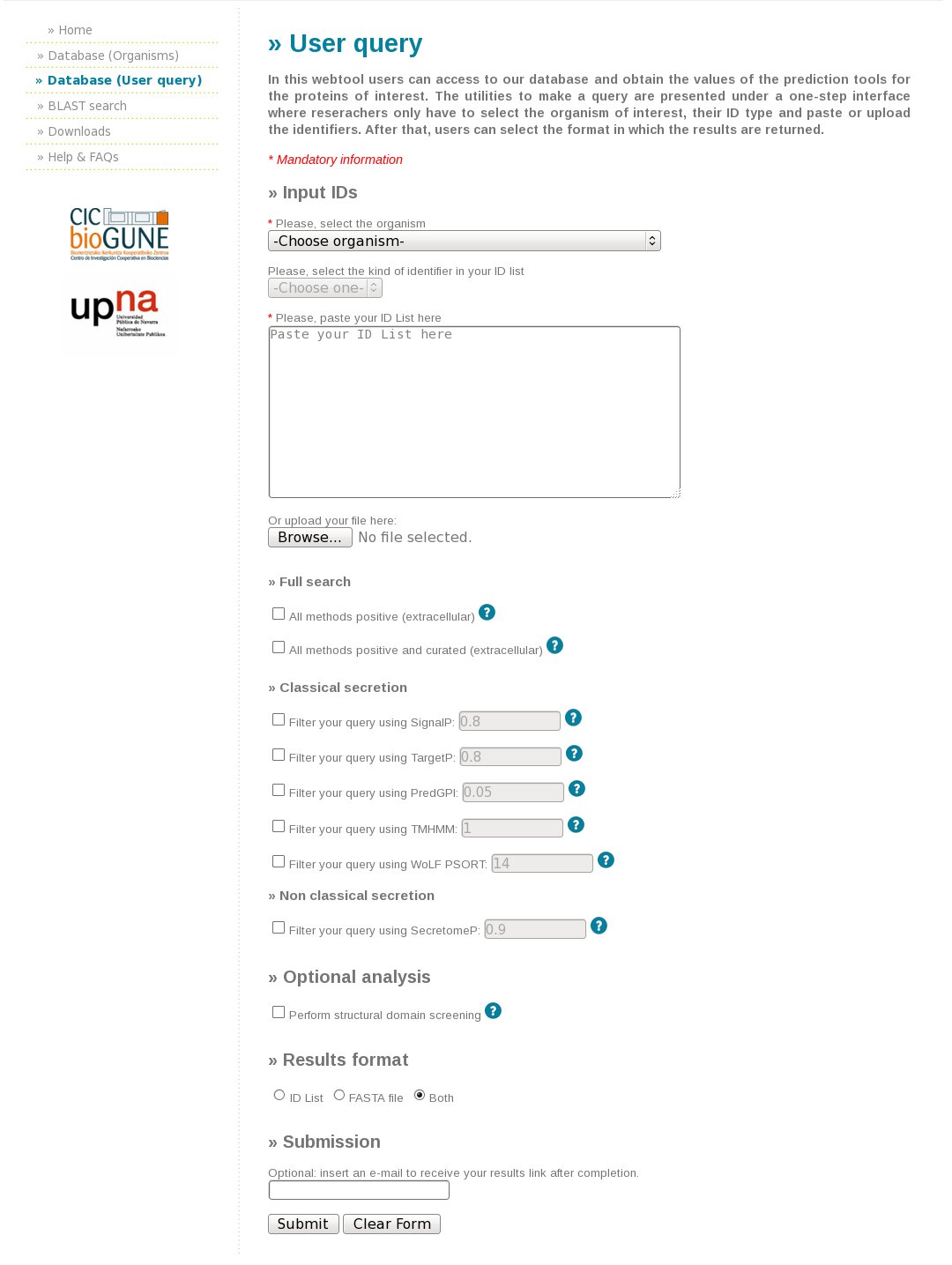
**

**Figure S5.** Local BLAST tool

**
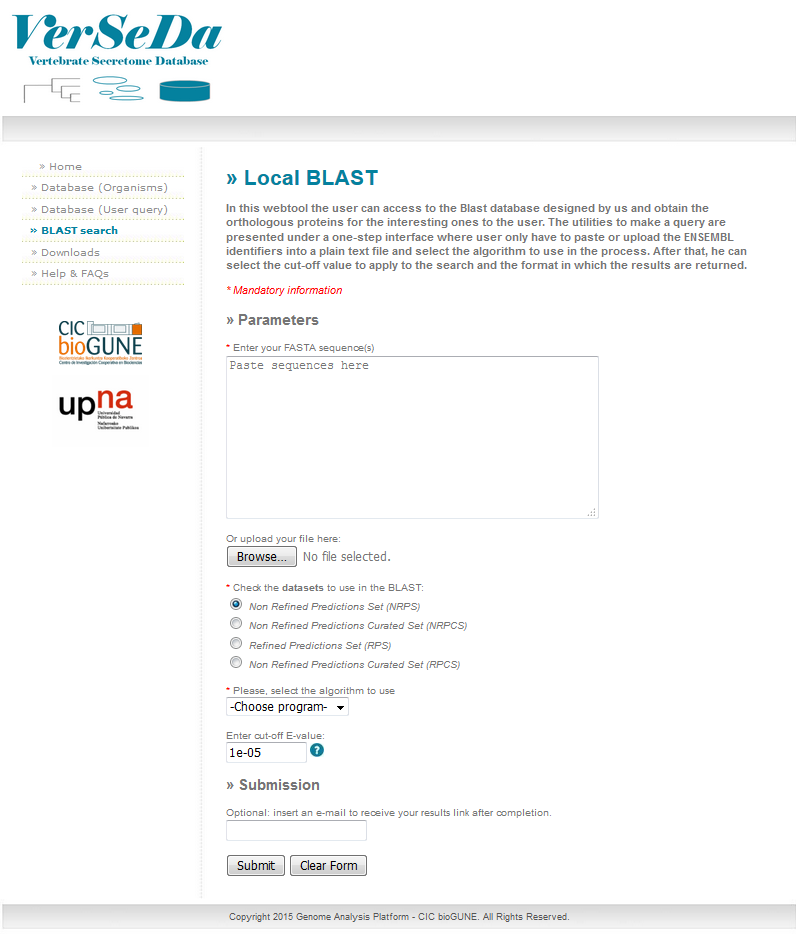
**

**Figure S6.** Database downloads section

**
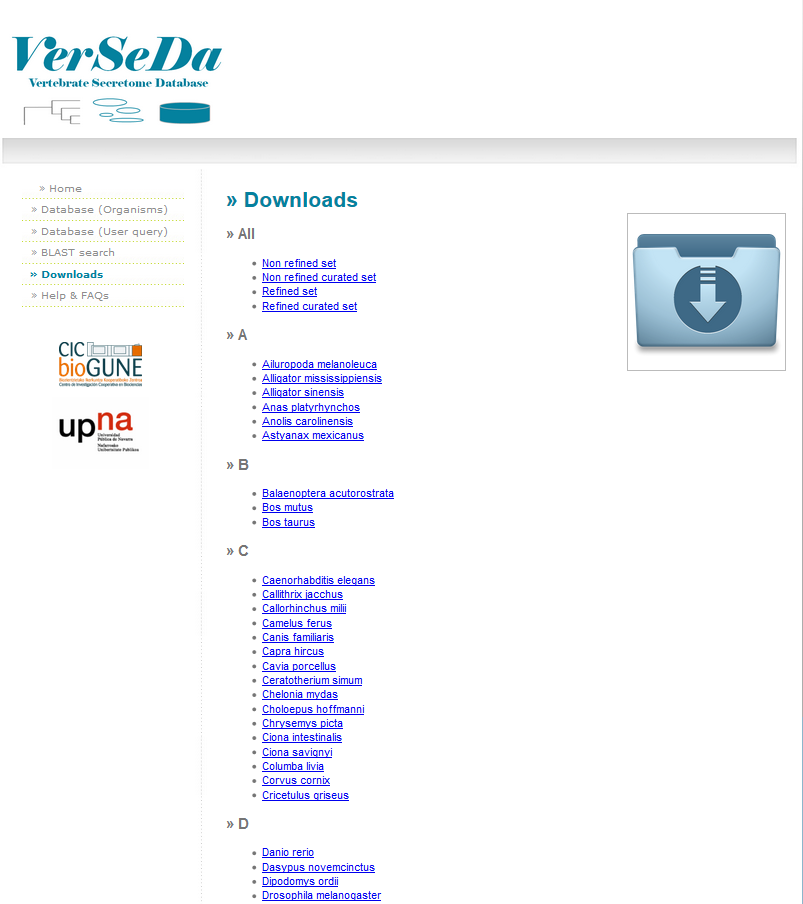
**
